# Oral Levetiracetam as Add-On Therapy in Refractory Neonatal Seizures 

**Published:** 2018

**Authors:** Mohsen MOLLAMOHAMMADI, Zeinab Sadat AMIRHOSEINI, Alireza SAADATI, Zahra PIRZADEH, Masoud HASSAN AMOUZADEH

**Affiliations:** 1 Neuroscience Research Center,Qom University of Medical Sciences, Qom, Iran.; 2 Neonatology-Perinatology, Pediatric Research Center, Qom University of Medical Sciences, Qom, Iran.; 3 Neurology Children Growth Research Center, Qazvin University of Medical Sciences, Qazvin , Iran.

**Keywords:** Neonatal seizure, Refractory Neonatal seizure, Oral levetiracetam, treatment

## Abstract

**Objective:**

Seizure is the most common signs of nervous system disease in neonates. The first line of treatments in neonatal seizures (after ruling out and emergency treatment of electrolyte imbalance and hypoglycemia) are phenobarbital and phenytoin. We aimed to evaluate drugs that are more effective on neonatal seizure.

**Materials & Methods:**

Patients admitted to neonatal wards & NICUs (level   IIa&  IIb) in Qom hospitals (2015-2017), central Iran with presentation of seizure, were enrolled in this clinical trial study. After ruling out electrolyte imbalance and hypoglycemia these neonates were managed by intravenous phenobarbital, then if no response was seen we added intravenous phenytoin and for remaining neonates with refractory seizure we applied oral levetiracetam as add on therapy. The study was registered as code number of IRCT2016051527896N1.

**Results:**

Initially, 245 neonates were enrolled. According to exclusion criteria, 12 cases were excluded, and phenobarbital was prescribed to the remaining patients. Out of these, 86 patients did not respond, and phenytoin was prescribed for them. Forty two patients who were not responding to phenytoin were finally treated with oral levetiracetam. Finally, 95.3% of seizures were controlled with oral levetiracetam but 4.7% were not cured.

**Conclusion:**

When the intravenous form of levetiracetam is not available and neonatal seizure does not respond to first line classic drugs, oral levetiracetam as add on therapy maybe effective.

## Introduction

Seizure is defined as a sudden changes in activity or behavior, sensory or autonomic function, due to paroxysmal electrical activity dysfunction of brain (1-3). Seizure in neonatal age has more prevalence than other period of life (4) that is 1–5 per 1, 000 live births (5). Annual prevalence is 1.8% to 8.6% (6-7), but in children is 0.5-1% (8). Variation in reported rates might be attributed to difficulty in diagnosis, different definitions of neonatal seizures and choosing a different enrolled criteria. 

There are different types of neonatal seizures including subtle (More common in preterm), clonic, tonic, spastic, and myoclonic. The incidence of neonatal seizure is different according to age of infants (1-4; 4-14 days; and more than 14 days). The most common leading cause is asphyxia or hypoxic-ischemic encephalopathy that involving 50%-75% of neonatal seizures (9-10). Other causes are infectious diseases, trauma, metabolic disorders, intra cranial &intra ventricular hemorrhage, and structural abnormalities of the brain (11). 

Neonatal seizure is one of the common causes of hospitalization that can be present with different patterns (12, 13). Depending on the etiology, different regime of antiepileptic drug could be needed (14-17). For example; if no structural defect detected, the anticonvulsants should be tapered during admission and in other conditions, long term-treatment is recommended (18). 

The nervous system of premature neonates, shows nonspecific response. More neonatal seizures are recovered completely and some of them may lead to transient or chronic complications (19). The brain grows and develops in first five years so frequent seizures not only effect on their development and learning capability but also leads to some structural changes in the brain (7). Thus, inhibition of seizure by an effective drugs is essential (20). 

Approach to neonatal seizure depends on underlying causes. The first line of treatment in neonatal seizure (after ruling out electrolyte imbalance and hypoglycemia) is phenobarbital followed by phenytoin (7). The potent anticonvulsive effects and low toxicity of phenobarbital, leads to using these drugs in neonatal seizures (20). Thus WHO introduced phenobarbital as a first line therapy in generalized, tonic, clonic and partial seizures (21-23). Some side effects for example are as follows: Sedative effects, behavioral disturbance and neuronal apoptosis of phenobarbital that are more than other similar drugs. But minimal systemic toxicity, long half-life and low cost are the main advantages versus other anticonvulsive drugs (24). In 35%-45% of seizures; phenobarbital and phenytoin as first line therapy were not effective, so other anti-convulsive agents should be added (25-29). 

New antiepileptic drugs (for example: topiramate, levetiracetam, lamotrigine, etc.) are recommended in resistant seizure in neonates. Levetiracetam is derived from pyrolidin. This drug is effective in neonatal seizure as add on or first line therapy. In addition, low efficacy towards serum protein, non-hepatic metabolism, absence of drug interaction and serious complications, and no neurotoxic effects, are other characteristics of this drug (27-29). 

According to above-mentioned features and because other first line drugs have many sides effects, in this study, levetiracetam is recommended in refractory neonatal seizure as add on therapy. So conducting this study would clarify the dark spots of its utilizing in patients. 

## Materials & Methods

Overall, 245 neonates presented with seizure in Qom hospitals (in neonatal wards &NICUs (level   IIa&  IIb)), were enrolled. Exclusion criteria were electrolyte imbalance and hypoglycemia. These neonates were managed by intravenous phenobarbital, then if no any response was seen, we added intravenous phenytoin, and for remaining neonates with refractory seizure, we applied levetiracetam as add on therapy. Because of unavailable of intravenous form, we used oral type. 

Neonates were treated with oral levetiracetam, administered in the form of gavage with initial dose of 10-20 mg/kg and gradually increased to 40-50 mg/kg if seizures was repeated (diagnosed according to clinical signs). Then the persistence of neonatal seizures, was monitored after drug administration. According to non-electrophysiological evaluation by subspecialists (in neonatology and pediatric neurology) about 95.3% of clinical seizures were completely controlled.

Informed consent was taken from the parents of the participants before administration of oral levetiracetam. Ethics Committee of the university approved the study. This prospective clinical trial study was registered as code number of IRCT2016051527896N1. 

Descriptive statistics (frequency, percentage) and analytical statistics (chi-square test) and SPSS ver. 20 (Chicago, IL, USA) were used to analyze the data. 

## Results

Initially, 245 neonates were enrolled in this study. According to exclusion criteria, 12 cases were excluded, and phenobarbital was prescribed to the remaining patients. Out of these, 86 patients did not respond, and phenytoin was prescribed for them. Forty two patients who were not responding to phenytoin; were finally treated with oral levetiracetam (Table 1). 

Influence of factors such as age, sex, type of delivery, gestational age, type and causes of seizure, CNS infections were measured on rate of response to oral levetiracetam. Fifteen patients (35.7%) were delivered by NVD & 27 patients (64. 3%) by C/S. Gestational age of 26 patients (61.9%) was term (over 37 weeks of gestation) and 16 patients (38.1%) preterm. Age of 35 patients (83.3%), was between a few hours to a week, 4 patients (9.5%) between 2 to 3 weeks, 2 patients (4.8%) between 3 to 4 weeks and 1 patient (2.4%) between 1 to 2 weeks. Twenty seven patients (64.3%) were male (Table 2). 

**Table 1:** The relationship between underline disease and response to levetiracetam

Underlying disease	Positive Response to levetiracetam	No Response to levetiracetam	Response to levetiracetam by increasing dose	*P* value
HIE	9(90%)	0	1(10%)	
Brain malformation	7(87. 5%)	1(12. 5%)		0. 838
IEM	6(75%)	1(12. 5%)	1(12. 5%)	
IVH	3(100%)	0	0	
Idiopathic	11(84. 6%)	0	2(15. 4%)	

No significant relationship was found between factors and response to oral levetiracetam. 

**Table 2:** The relationship between response to treatment by oral levetiracetam and demographic data

**Demographic data**	Positive Response to levetiracetam	No Response to levetiracetam	Response to levetiracetam by increasing dose	*P* value
**Gestational age (weeks)**				
-Term >=37 -Preterm <37	21(58. 3%) 15(41. 7%)	1(50%) 1(50%)	4(100%) 0(100%)	0. 382
**Sex**				
-Male -Female	23(63. 9%) 13(36. 1%)	2(100%) 0(0%)	2(50%) 2(50%)	0. 657
**Mode of delivery**				
-NVD(vaginal delivery)	13(36. 1%)	0(0%)	2(50%)	0. 654
-C/S (cesarean section )	23(63. 9%)	2(100%)	2(50%)	
**Post-natal age(days)** -< 7	30(83. 3%)	2(100%)	3(75%)	
8-14	0(0%)	0(0%)	1(25%)	
15-21	4(11. 1%)	0(0%)	0(0%)	0. 207
22-28	2(5. 6%)	0(0%)	0(0%)	
**CNS infection**				
Infection No infection	0(0%) 36(100%)	0(0%) 2(100%)	1(25%) 3(75%)	0. 147
**Type of seizure**				
-Myoclonic -Tonic -Subtle -Spastic -Clonic -Mixed Form	8(22. 2%) 2(5. 6%) 9(25%) 5(13. 9%) 9(25%) 3(8. 3%)	2(100%) 0(0%) 0(0%) 0(0%) 0(0%) 0(0%)	1(25%) 0(0%) 0(0%) 1(25%) 0(0%) 2(50%)	0. 177

Underlying cause of seizure in 10 patients (23. 8%) was HIE (hypoxic-ischemic encephalopathy) and in 8 patients (19%) - brain malformation, in 8 patients (19%) – IEM (inborn errors of metabolism) in 3 patients (7.1%) IVH (intra ventricular hemorrhage) and 13 patients (30.9%) was idiopathic. 

## Discussion

This clinical trial study showed that oral levetiracetam (in case of unavailability of intravenous form) is useful in controlling neonatal seizures. About 94.5% of neonates that did not respond to first line therapy, completely cured. This study investigates new aspects of the subject because had used levetiracetam after phenobarbital and phenytoin in refractory seizure as oral type and based on the best knowledge of authors, not applied in any other study as oral type. The relationship between basic factors such as age, sex, type of delivery, gestational age, type & causes of seizures and CNS infection with response to treatment were evaluated, but no significant relationships were found. Oral levetiracetam is an effective drug in treatment of neonatal seizures in our study. 

In a study on 38 neonates with seizures without hypoglycemia, hypomagnesaemia, hypocalcaemia and pyridoxine deficiency, intravenous levetiracetam was used as first-line therapy with more effectiveness than phenobarbital, without any side effects (30). In our study, the effect of oral levetiracetam has been evaluated in neonatal seizures that was not responding to other anticonvulsant therapy and proven to be effective without any side effects. Khano et al. studied 22 neonates with seizures, treated with intravenous levetiracetam at the dose of 10 to 50 mg/kg. In all cases, seizures were totally subsided after 72 h and 86% of patient were discharged with oral levetiracetam (31). In our study, in order to unavailability of intravenous levetiracetam, we used the oral form of this drug and received to the same results. 

A study was conducted on the 8 neonates with resistant seizures to first line anticonvulsant therapy, then they were treated by intravenous levetiracetam. As a result, 6 of them were completely cured, one patient did not respond and one had a partial response to the treatment (32). In our study, the efficacy of oral levetiracetam was evaluated by total cure of 36 neonates out of 42, with initial dosage, 2 neonates were cured by increasing the dose and 4 patient were not responding to oral therapy. 

**In conclusion**, in several studies; intravenous form of levetiracetam was evaluated with favorable effect without any complication, and in emergency condition it was first line of therapy. We showed in special conditions, oral levetiracetam could be used as an additional therapy with good effect in control of resistant neonatal seizures. Further studies are needed to reach exact conclusion and better results. 
